# Current application status of non-invasive brain stimulation techniques in Alzheimer’s disease: a bibliometric analysis

**DOI:** 10.3389/fnagi.2025.1585885

**Published:** 2025-09-25

**Authors:** Shan Cong, Meng Wang, Long Yan, Li Sun, Bowen Zheng, Jinying Xie, Tao Yu, Yulin Qian

**Affiliations:** 1 The First Teaching Hospital of Tianjin University of Traditional Chinese Medicine, Tianjin, China; 2 National Clinical Research Center for Acupuncture and Moxibustion, Tianjin, China; 3 Graduate Department, Tianjin University of Traditional Chinese Medicine, Tianjin, China; 4 Tianjin Xiqing District Traditional Chinese Medicine Hospital, Tianjin, China

**Keywords:** Alzheimer’s disease, non-invasive brain stimulation, transcranial magnetic stimulation, bibliometric analysis, CiteSpace

## Abstract

**Objective:**

Alzheimer’s disease (AD) poses a significant global public health challenge. Non-invasive brain stimulation (NIBS) has emerged as a promising therapeutic strategy and constitutes a rapidly evolving research domain for AD intervention. This study aims to synthesize recent advancements in NIBS technologies for AD through comprehensive knowledge mapping. By mapping the research landscape, identifying key trends, and analyzing collaborative networks, we seek to explore emerging frontiers and translational potential of NIBS in AD research, thereby informing evidence-based clinical practice.

**Methods:**

Using the Science Citation Index Expanded (SCI-E) of Web of Science Core Collection (WOSCC) database. The analysis included an evaluation of publication trends, journal distribution statistics, country/region and institutional collaboration networks, author and co-cited author networks, co-citation document networks, as well as keywords and research hotspot analysis. Then CiteSpace, GraphPad Prism, VOSviewer, Microsoft Excel and NoteExpress were used for follow-up bibliometric analysis.

**Results:**

A total of 632 studies were included in this study. Research on NIBS applications in AD peaked during 2020–2021. The predominant journals disseminating NIBS-AD research were Journal of Alzheimer’s Disease, Frontiers in Aging Neuroscience, and Clinical Neurophysiology. Italy, China, and the United States led in research contributions during this period. At the institutional level, Harvard Medical School and the University of Brescia published the most. There are 529 authors in this field, among which Professor Giacomo Koch maintains a continuous academic leadership position. Keyword analysis revealed high-frequency terms, “Alzheimer’s disease,” “transcranial magnetic stimulation,” and “mild cognitive impairment.” “Impairment” and “non-invasive brain stimulation” emerged as citation burst terms from 2022 onward, signaling current investigative priorities centered on NIBS-induced cognitive modulation, therapeutic target selection, and underlying neurophysiological mechanisms.

**Conclusion:**

This study comprehensively reviews current research status, hotspots and trends of NIBS in AD. The results suggest that researchers should focus on the cognitive impact of NIBS technology on AD patients, the best therapeutic targets and potential mechanisms. Strengthening global collaboration among international, institutional and scientific researchers should be promoted to promote the in-depth development of this field.

## 1 Introduction

Alzheimer’s disease (AD) is a prevalent neurodegenerative disorder, which will affect 150 million individuals worldwide by 2050, positioning AD as a critical public health challenge (De Paolis et al., 2024; Yan et al., 2024). Despite substantial research focused on elucidating the pathogenesis of AD, identifying biomarkers for early diagnosis, and developing effective treatments, the intricate pathophysiological mechanisms underlying the disease remain incompletely understood. Current medication therapeutic mainly include acetylcholinesterase inhibitors (e.g., Donepezil, Galantamine), NMDA receptor antagonists (e.g., Memantine), and anti-amyloid-*β* monoclonal antibodies (e.g., Lecanemab, Donanemab, Aducanumab), these drug intervention limited efficacy, and prone to adverse reactions (Liang et al., 2024; Haque and Levey, 2019), which seriously impacts the quality of life of middle-aged and elderly adults. To date, no treatment capable of achieving complete recovery has been developed (Wojtecki et al., 2024; Petrovskaya et al., 2023), highlighting the urgent need to develop innovative therapeutic strategies for treating AD.

Non-invasive brain stimulation (NIBS) has emerged as a safe neuromodulatory approach with potential applications in neurodegenerative diseases, and has become a major research focus in various fields in recent years (Yang et al., 2024). Techniques such as transcranial magnetic stimulation (TMS), transcranial direct current stimulation (tDCS), transcranial alternating current stimulation (tACS), transcutaneous auricular vagus nerve stimulation (taVNS), photobiomodulation, and transcranial ultrasound/pulse stimulation have demonstrated promise in modulating neural excitability and plasticity. Among these, TMS (Lefaucheur et al., 2020; Georgopoulou et al., 2024) and tDCS (Teselink et al., 2021; Chen et al., 2022) are the most widely studied. Clinical evidence suggests NIBS may enhance early AD diagnosis and cognitive rehabilitation by influencing neuroplasticity (Kim S. K. et al., 2024; Kim T. et al., 2024; Manippa et al., 2024; Elahi and Frechette, 2023; Benussi et al., 2022; Yao et al., 2022). However, its long-term efficacy and precise mechanisms require further exploration.

Bibliometric analysis, supported by tools like CiteSpace, enables quantitative and qualitative evaluation of research trends, hotspots, and collaborative networks within a field (Zhang et al., 2023). With its robust analytical capabilities, it enables researchers to systematically map research trajectories and identify emerging research frontiers, making it an invaluable tool for advancing scientific discovery (Zhang et al., 2024). Current bibliometric studies on AD predominantly focus on analyzing research hotspots and influencing factors (Liu et al., 2019; Shi et al., 2022; Yue et al., 2022). While NIBS ameliorates cognitive impairments in AD by enhancing neuroplasticity, strengthening neurovascular coupling, and attenuating neuroinflammation (Pievani et al., 2017; Lefaucheur et al., 2020; Ferrucci et al., 2008), there remains a significant scarcity of integrative bibliometric analyses comparing the therapeutic efficacy of different NIBS modalities. This study employs CiteSpace to analyze NIBS-AD research, providing insights into current advancements, emerging hotspots and trends. It also lays the groundwork for future research and clinical applications, exploring emerging frontiers and translational potential of NIBS in AD research, thereby, informing evidence-based clinical practice.

## 2 Data and methods

### 2.1 Data collection

**Figure 1:**
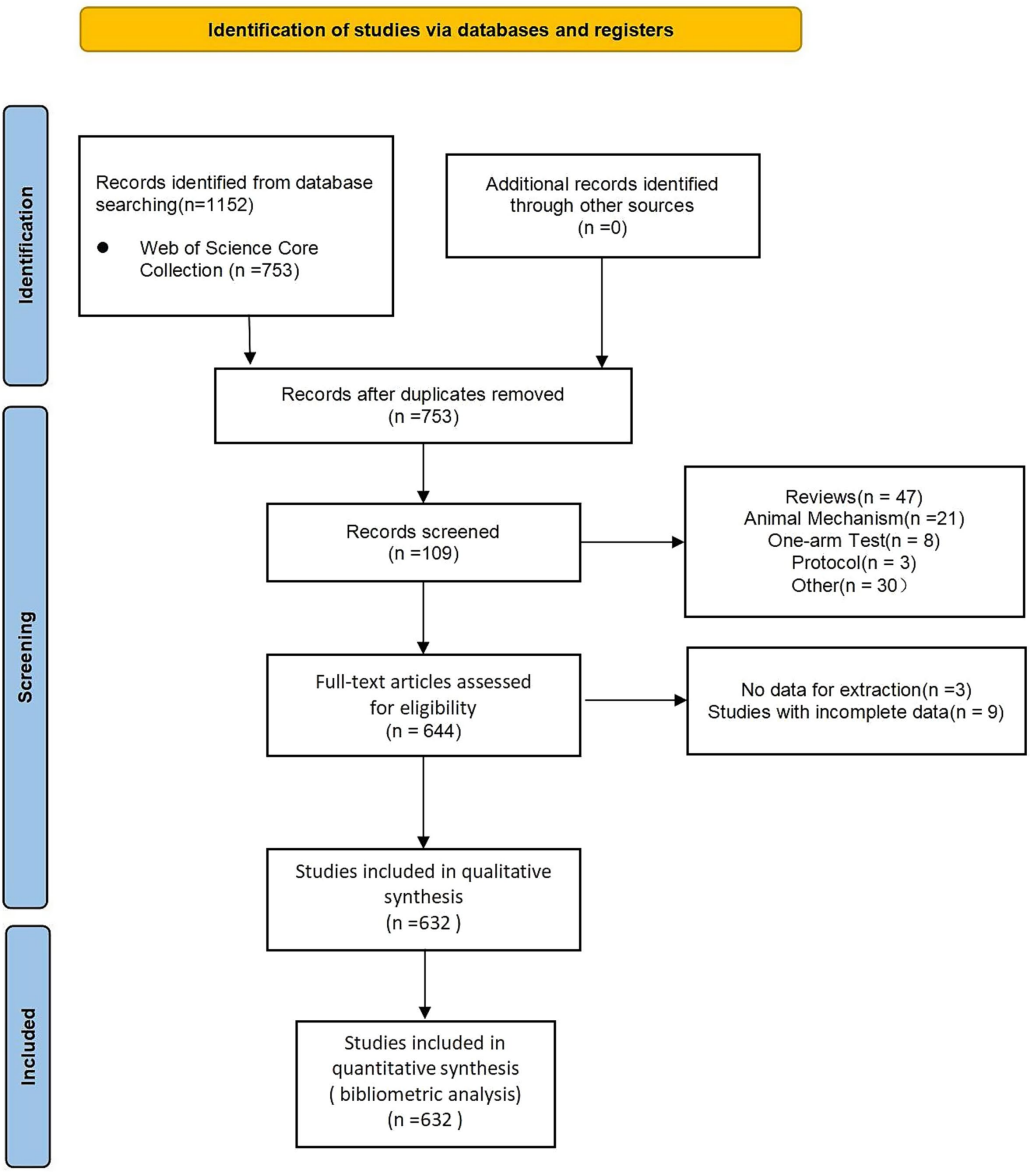
Flow chart of the literature screening.

The data utilized in this study were retrieved from the Science Citation Index Expanded (SCI-E) within the Web of Science Core Collection (WOSCC) database. The search strategy was designed as follows: TS = (“Alzheimer’s disease” OR “Alzheimer disease”) AND TS = (“non-invasive brain stimulation” OR “non-invasive neuromodulation technology” OR “transcranial direct current stimulation” OR “transcranial magnetic stimulation” OR “transcranial ultrasound stimulation” OR “transcutaneous auricular vagus nerve stimulation” OR “transcranial alternating current stimulation” OR “transcranial pulse stimulation”). The time span was set from the establishment of the database to November 27, 2024, and the language was restricted to English. Following the initial search, all retrieved publications were systematically screened by reviewing their titles and abstracts. Publications that did not meet the inclusion criteria were excluded. The process was independently performed by two reviewers, and the third reviewer reviewed the studies with ambiguity. The included studies were randomized controlled trials, cohort studies, prospective studies, etc., covering search-based AD and NIBS. The screening process is shown in Figure 1.

### 2.2 Analytical tool

The analysis and visualization of results in this study were conducted using the following software tools: CiteSpace 6.3.3.0, GraphPad Prism 10.4.1, VOSviewer 1.6.20, Microsoft Excel 2021, and NoteExpress 1.0.0.0. The analytical framework encompassed multiple dimensions, including publication trends, journal distribution statistics, country/region and institutional collaboration networks, author and co-cited author networks, co-citation document networks, as well as keywords and research hotspot analysis. These tools and methods were employed to provide a comprehensive and systematic overview of the research landscape.

Following data import into CiteSpace and VOSviewer. The VOSviewer was set to the maximum number of countries per document to 25, and the minimum number of documents per country to>5. The CiteSpace was set to have a time range spanning 1999–2024 with 1-year slicing intervals. Key selection parameters included a g-index threshold (k = 25), look-back factor (LRF = 2.0), maximum links per node (L/N = 10), minimum burst duration (LBY = 8), and edge weight scaling exponent (e = 2.0). Network optimization employed Pathfinder pruning with sliced network simplification to generate topology-refined diagrams. There are several bibliometric indicators that need special attention, including centrality and outbreak intensity. Centrality, quantifying node bridging significance within networks, visually encoded by purple rings where thicker annuli denote higher betweenness centrality values, identifying pivotal knowledge hubs. Burst strength, detecting transient research hotspots via Kleinberg’s state-transition algorithm, which identifies significant frequency spikes in keywords during specific periods. These indicators collectively reveal structural pivots and temporal trend shifts within the knowledge domain.

## 3 Results

### 3.1 Publication years, journals, and categories

**Figure 2:**
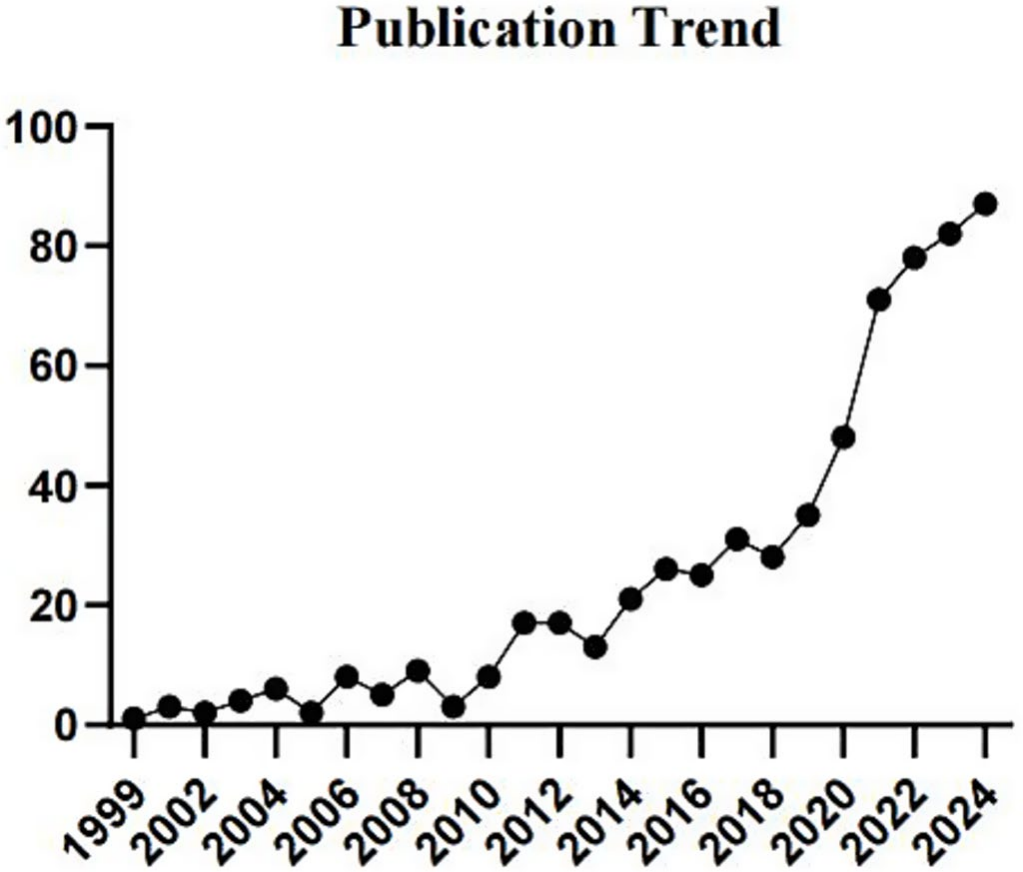
Trend diagram of journal publication.

A total of 632 NIBS-AD studies were identified in the WOSCC database. Publication trends (Figure 2) revealed low interest in NIBS-AD applications prior to 2000, followed by a surge during 2020–2021. Following 2021, the rate of publication growth decelerated. However, the annual number of publications continues to rise, indicating sustained research activity in the field.

Table 1 lists the top 10 journals by publication output for NIBS-AD research, along with their corresponding impact factors (updated to 2023 via the WOS database), providing critical references for researchers in this field. The Journal of Alzheimer’s Disease ranked first, publishing 44 NIBS-AD-related articles, with an annual output exceeding 700 articles and an impact factor of 3.4. The second-ranked journal, Frontiers in Aging Neuroscience, contributed 37 NIBS-AD publications. In 2024, the journal published over 1,400 articles, representing a notable rise compared to 2023, with its publication output remaining on an upward trajectory. The journal holds an impact factor of 4.1, reflecting its growing influence in the field of aging neuroscience. Clinical Neurophysiology ranked third with 27 NIBS-AD publications, maintaining an average annual output of ~85 articles over the past decade and an impact factor of 3.7. It is evident that articles focusing on NIBS-AD are more commonly featured in journals specializing in Alzheimer’s disease, as well as in professional periodicals dedicated to neurological disorders. And the impact factors are mostly concentrated in 3–4 points.

**Table 1:** Statistical table of the top 10 journals published in this field.

Journals	Impact factor	Number of published
Journal of Alzheimer’s Disease	3.4	44
Frontiers in Aging Neuroscience	4.1	37
Clinical Neurophysiology	3.7	27
Brain Stimulation	7.6	27
Journal of Neural Transmission	3.2	16
Frontiers in Neuroscience	3.2	14
Frontiers in Human Neuroscience	2.4	14
Alzheimer’s Research & Therapy	8.0	13
Neurobiology of Aging	3.7	13
Frontiers in Neurology	2.7	13

**Figure 3:**
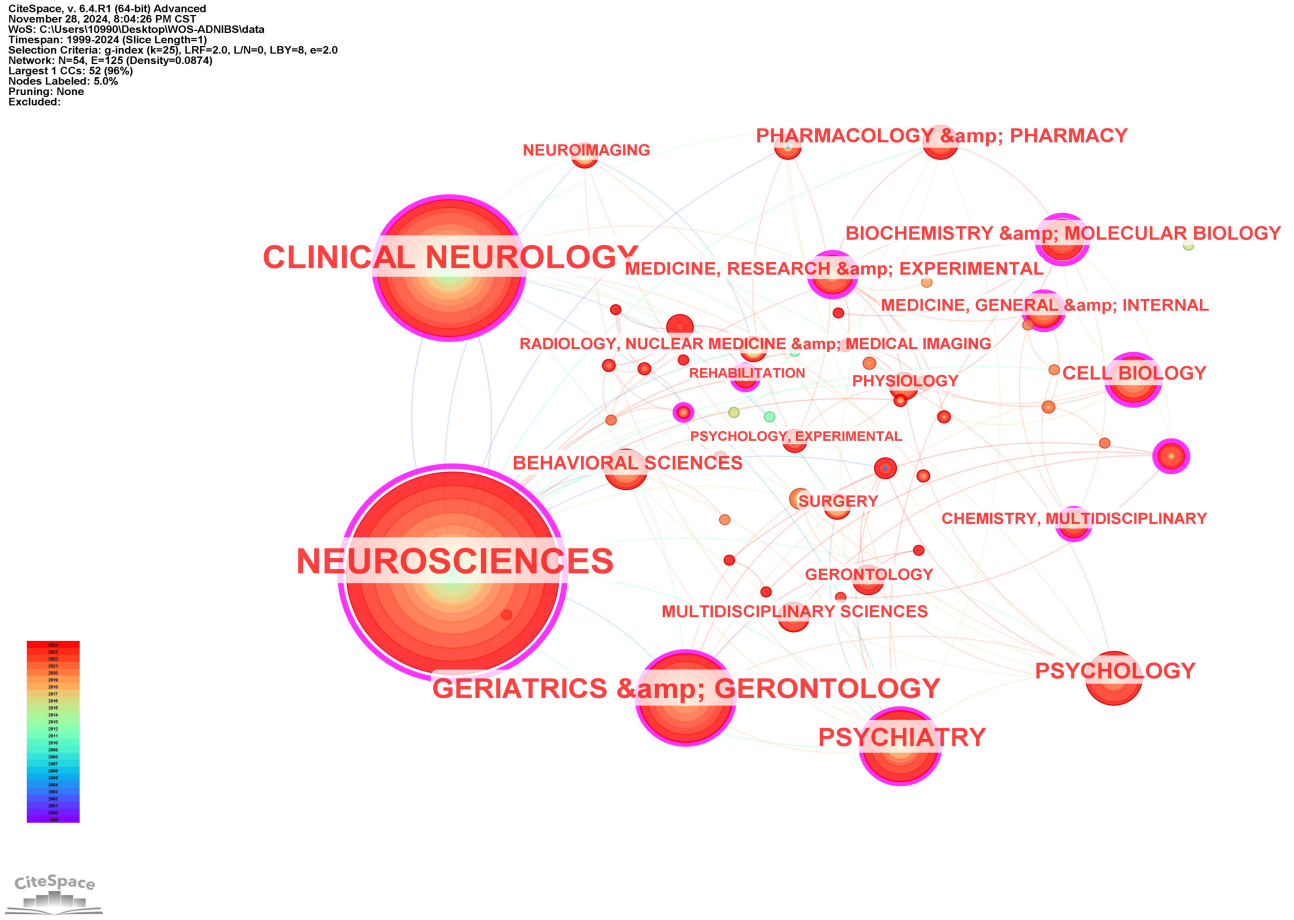
Subject categories co-occurrence map.

The distribution of the top 10 research categories in this field reveals that Neuroscience leads with 421 publications, followed by Clinical Neurology (234 publications), collectively accounting for over half of the total. This indicates that NIBS-AD research primarily focuses on fundamental and clinical aspects related to the nervous system. Categories such as geriatric medicine and gerontology (92 publications) and psychiatry (68 publications) also hold significant proportions, reflecting close connections with interdisciplinary fields like aging and mental health. The overall landscape demonstrates a core focus on neuroscience and clinical neurology while extending into multidisciplinary intersections (as shown in Figure 3).

**Figure 4:**
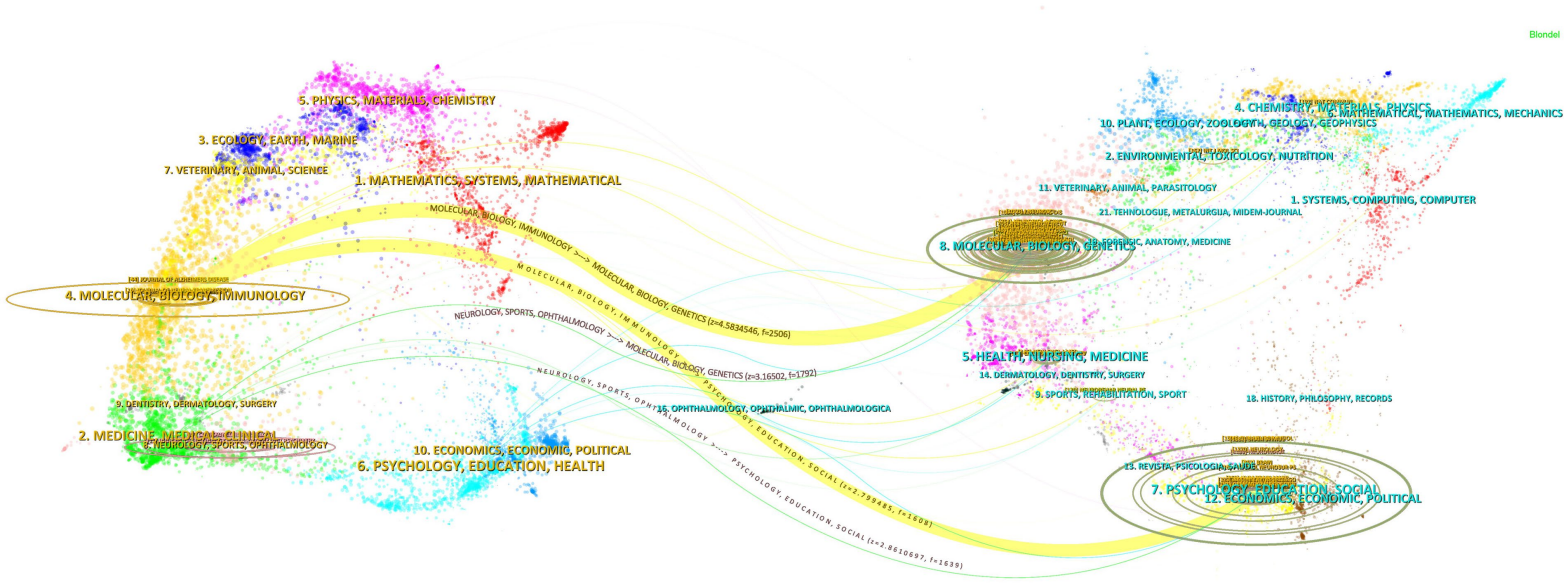
Dual graph superposition of journals.

From the dual-map overlay analysis of journals (Figure 4), the left region represents the set of citing journals, while the right region represents the set of cited journals. The colored pathways connecting the two regions illustrate citation relationships across diverse research fields. Notably, two prominent yellow citation pathways highlight that research published in molecular, biological, and immunology journals predominantly cites content from molecular, biology, and genetics research journals, as well as psychology, education, and social research journals. This reflects the foundational role of basic biomedical sciences in AD research, while highlighting its multidisciplinary focus on patients’ cognitive-behavioral profiles and psychosocial functioning. Crucially, it demonstrates substantive interconnections across research domains, thereby providing a broader perspective for analyzing and understanding the complex pathophysiological mechanisms and clinical intervention strategies of AD.

### 3.2 Analysis of the most productive countries/regions and institutions

**Figure 5:**
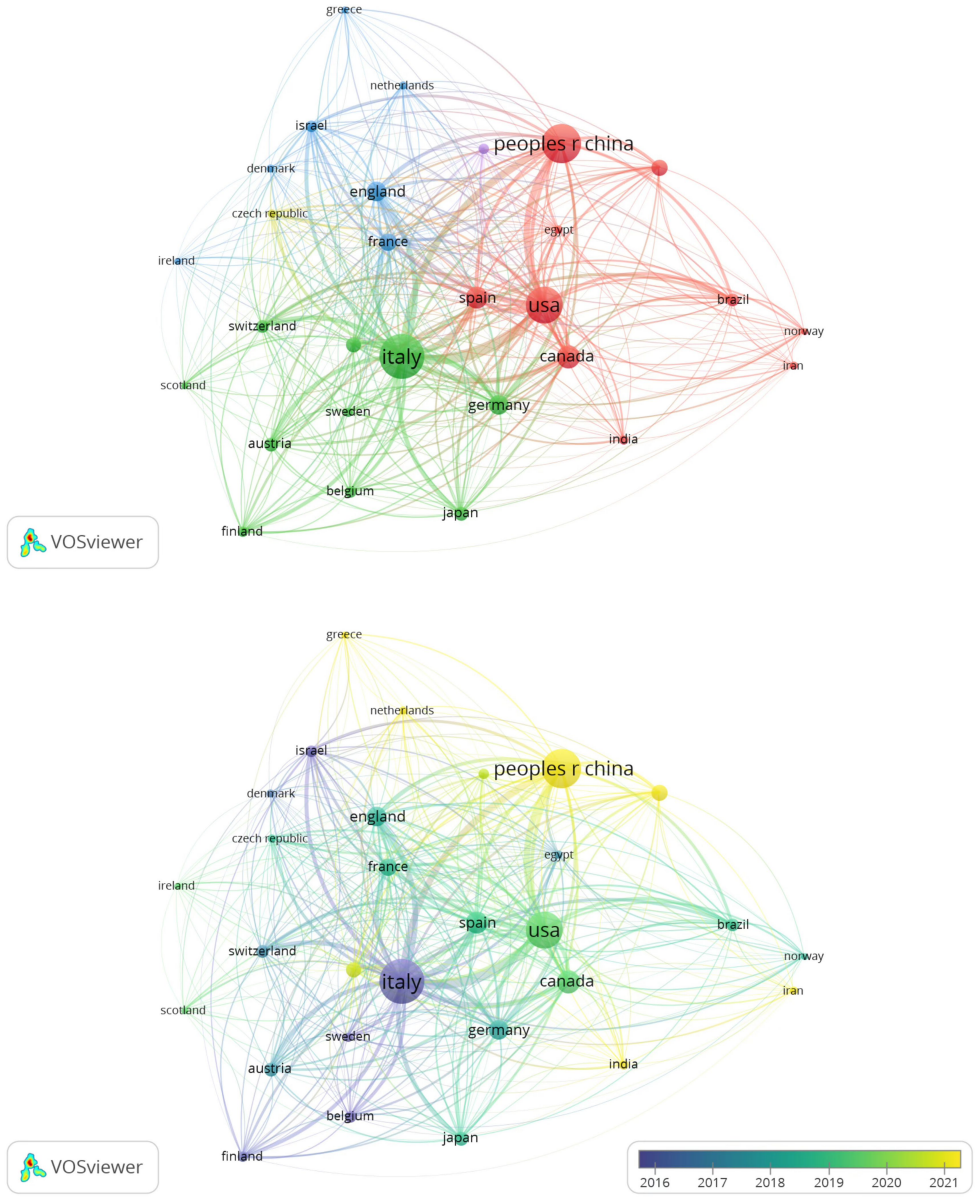
Country co-occurrence map.

**Figure 6:**
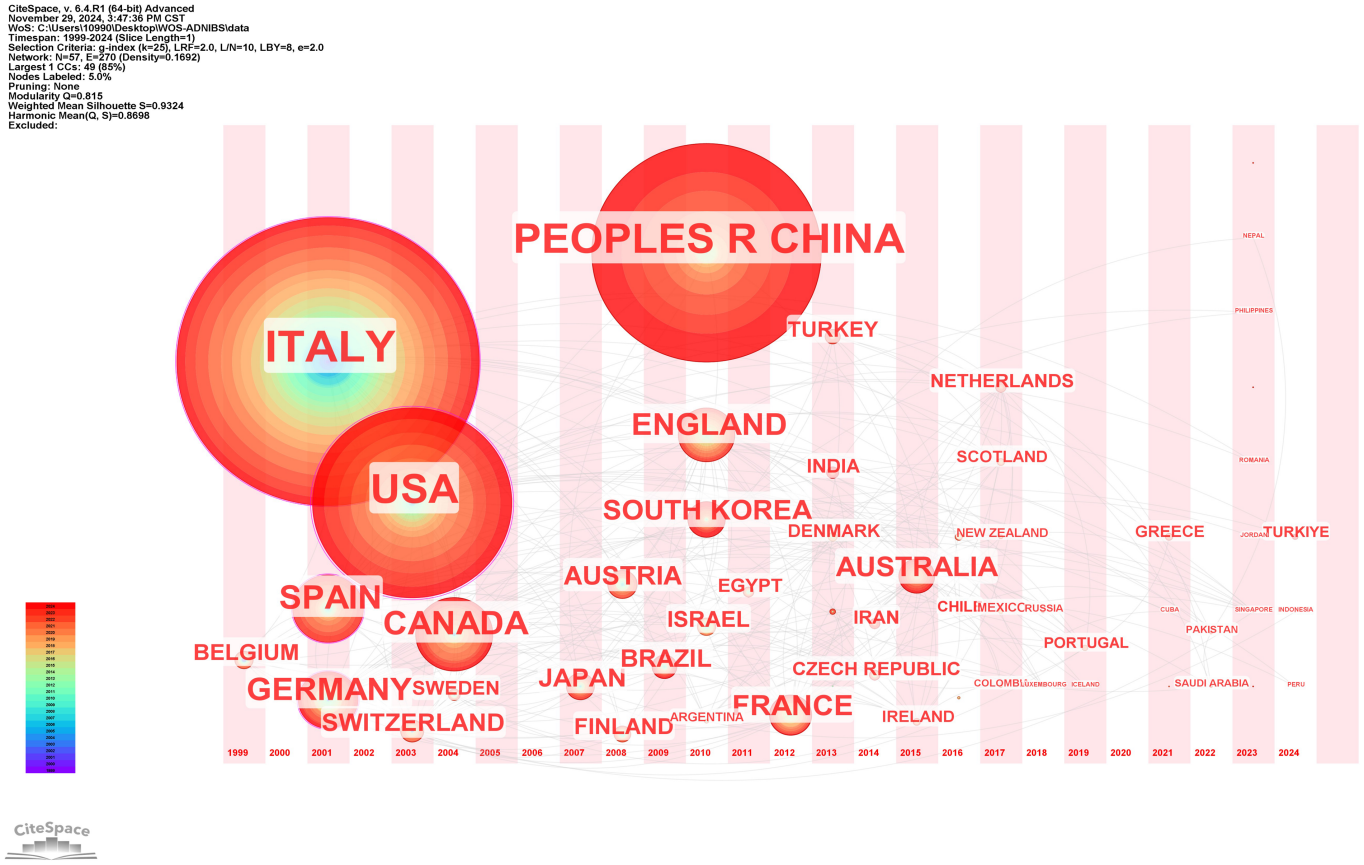
Time-zone diagram of country co-occurrence.

A total of 29 countries met the inclusion criterion of publishing more than five articles, which were categorized into five clusters according to their collaborative intensity. The top five countries contributing the most to the research output were Italy (centrality = 0.16), China (centrality = 0), the United States (centrality = 0.34), Canada (centrality = 0.06), and Spain (centrality = 0.11). The publications from the United States and Italy exhibited the highest citation centrality, reflecting their relatively significant academic impact. China initiated its research on this field in 2010, despite a substantial number of publications, the citation frequency of these studies remains relatively low, thereby limiting their academic influence. This likely stems from constrained collaborative engagement between Chinese researchers and globally leading institutions, limiting international visibility and discourse of their findings, consequently diminishing citation impact within the scholarly community (Figures 5, 6).

**Figure 7:**
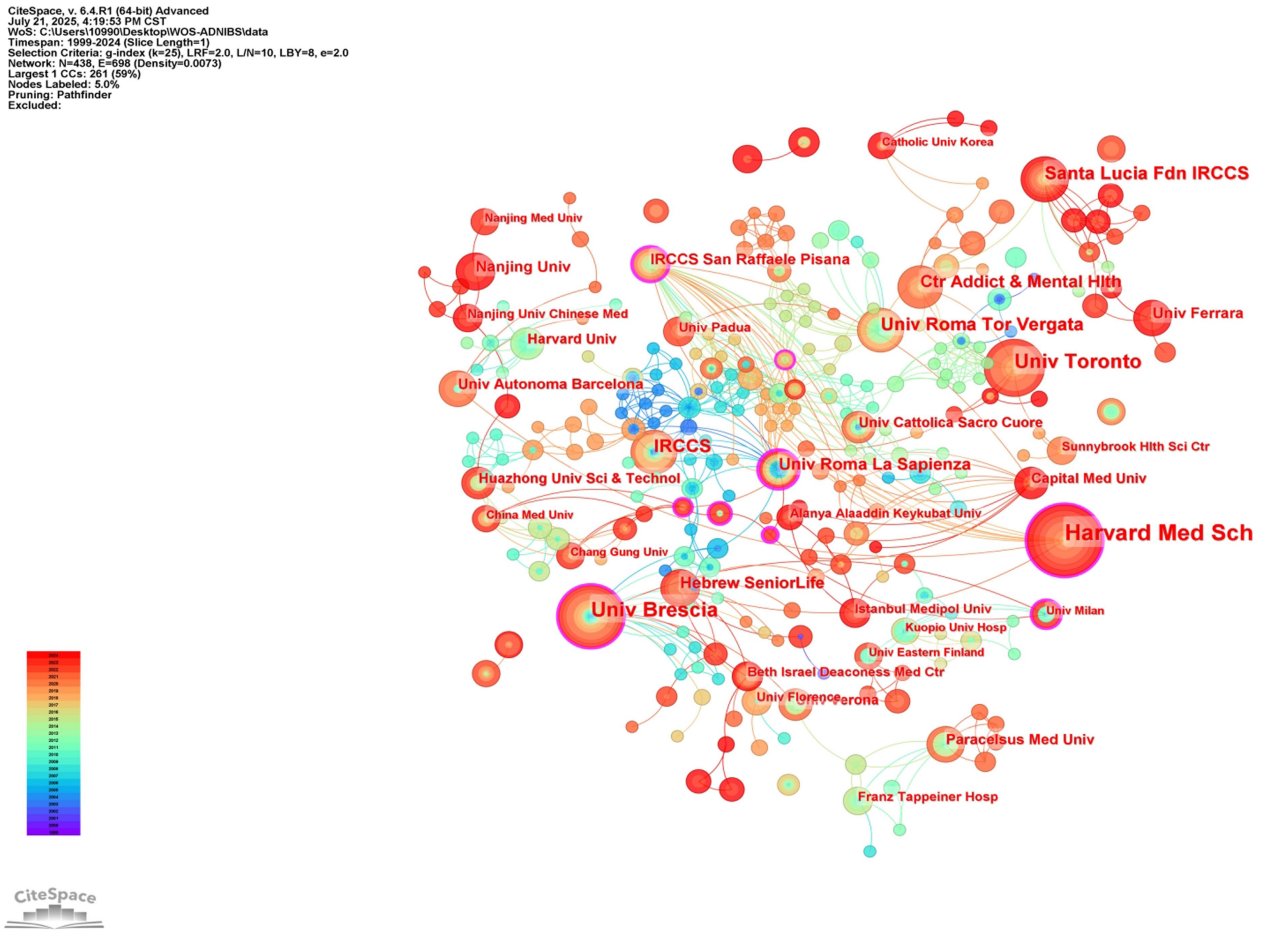
Institution co-occurrence map.

Table 2 presents the top ten institutions with the highest number of published papers in this field. Centrality is often associated with an institution’s connectivity within collaborative networks. While Harvard Medical School (centrality = 0.07) leads in paper publications, Sapienza University of Rome (centrality = 0.37) ranks second with the highest citation centrality. This may be attributed to the institution’s extensive collaborations with internationally renowned entities, which position its research achievements as pivotal nodes in collaborative networks, thereby demonstrating significant academic influence. Figure 7 displays a global collaborative network map among these institutions.

**Table 2:** The ten most influential organizations.

Count	Centrality	Year	Institution
41	0.11	2017	Harvard Med Sch
29	0.14	2006	Univ Brescia
25	0.09	2017	Univ Toronto
17	0.06	2014	Santa Lucia Fdn IRCCS
16	0.09	2009	Univ Roma Tor Vergata
16	0.01	2003	IRCCS
15	0.05	2015	Ctr Addict & Mental Hlth
13	0.37	2003	Univ Roma La Sapienza
11	0.07	2020	Hebrew SeniorLife
10	0.01	2023	Nanjing Univ

### 3.3 Analysis of authors and co-cited authors

**Figure 8:**
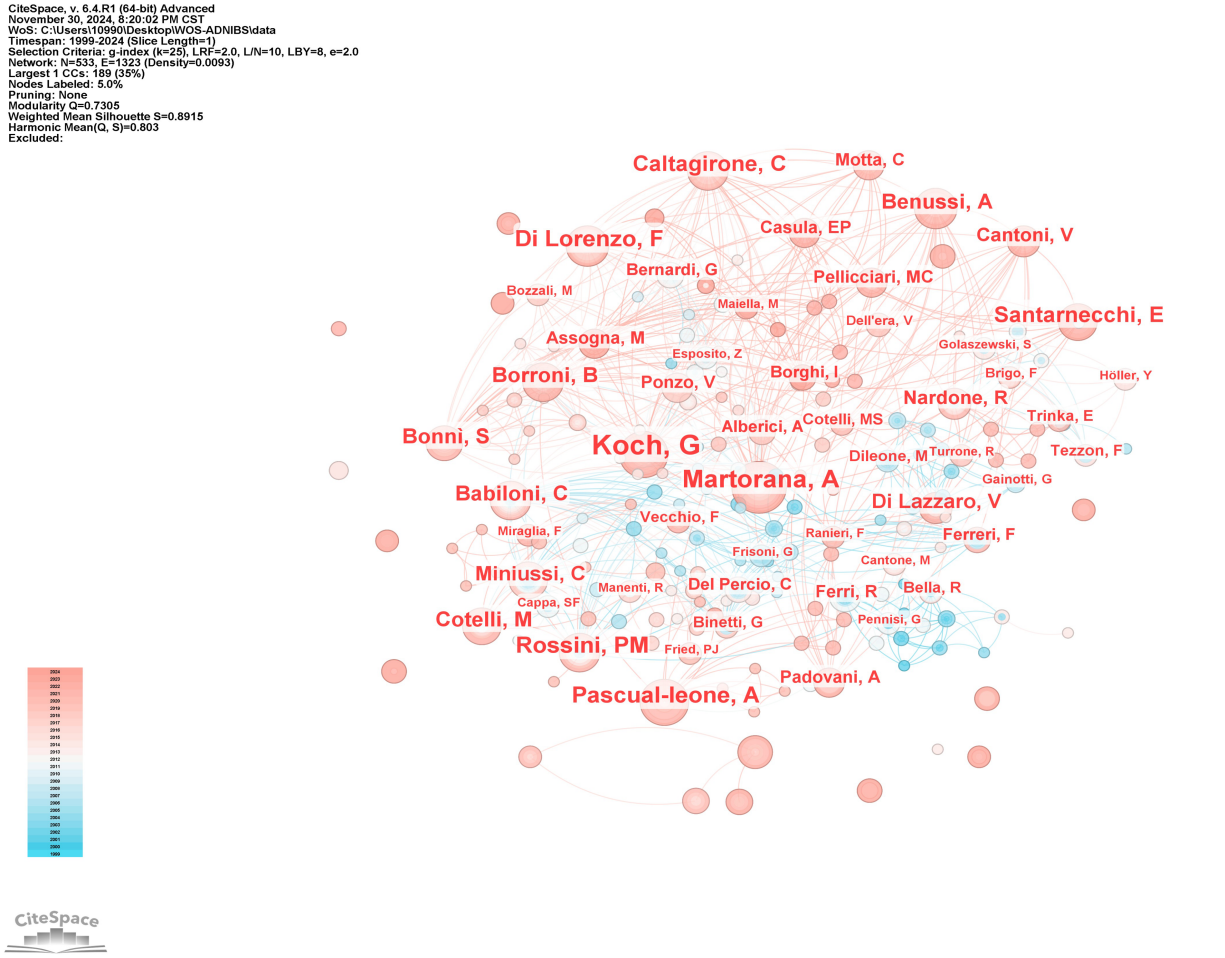
Author co-occurrence map.

**Figure 9:**
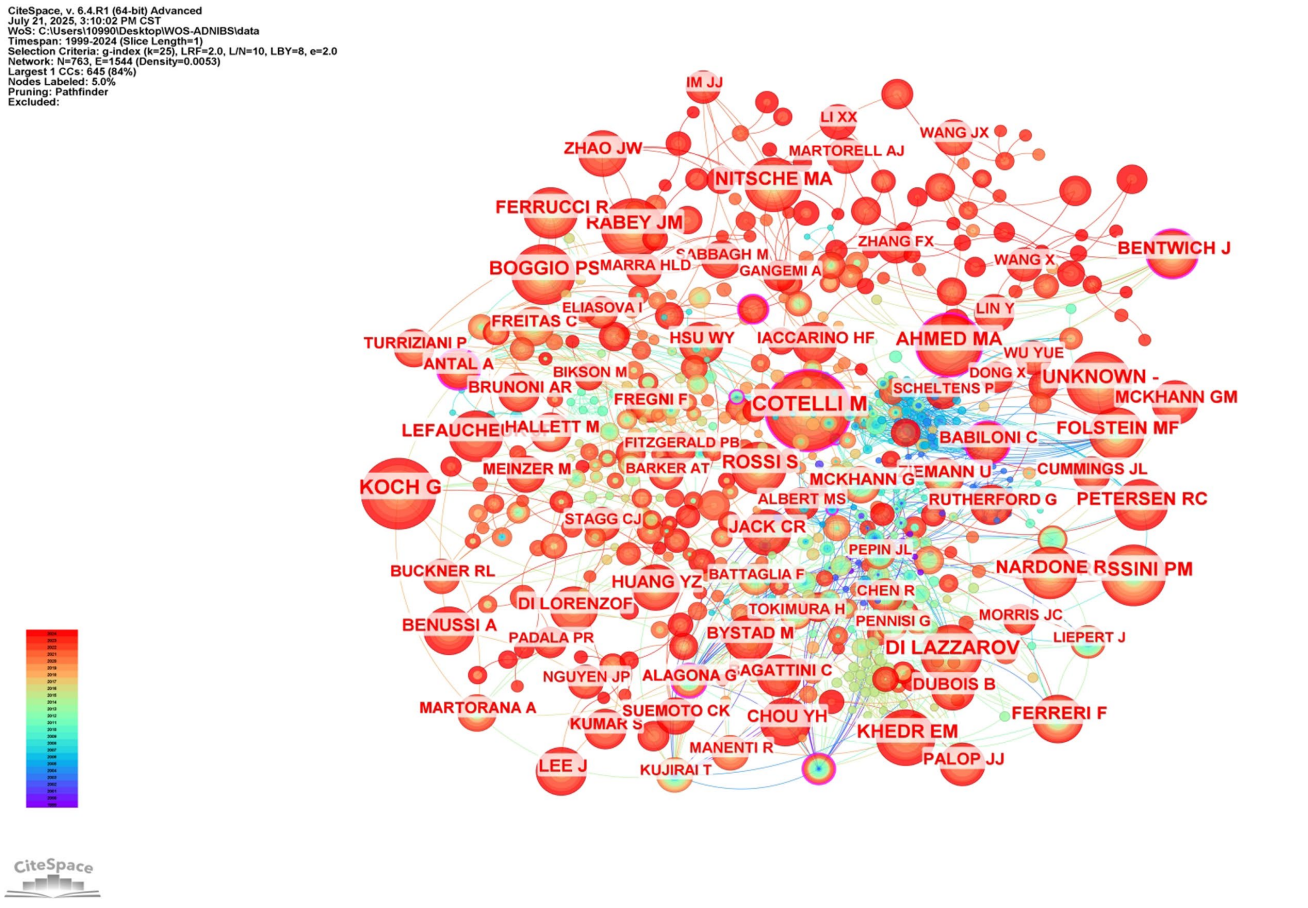
Authors co-cited map.

This study identified 529 authors contributing to NIBS-AD research. Figure 8 maps global collaboration networks, while publication metrics reveal top scholars: Giacomo Koch (centrality = 0.05) leads with 39 publications, followed by Alessandra Matolanana (centrality = 0.01) with 28 publications, Francesco Di Lorenzo (centrality = 0.01) and Paolo Maria Rossini (centrality = 0.04) each have 21 publications, and Alvaro Pasquale-Leone (centrality = 0.02) with 20 publications. These high-output researchers constitute pivotal contributors. Notably, Vincenzo Di Lazaro (14 papers, centrality = 0.06) has published less than top authors, but has the highest centrality, highlighting his major contribution to the field. Figure 9 demonstrates author co-citation patterns, with Maria Cotelli (207 co-citations, centrality = 0.18), Giacomo Koch (169 co-citations, centrality = 0.07), and Vincenzo Di Lazzaro (147 co-citations, centrality = 0.05) dominating influence metrics. Koch’s dual prominence in productivity and connectivity solidifies his central role in NIBS-AD scholarship. Koch, G consistently leads in both co-occurrence and co-citation rankings. This dual trait of “high output coupled with strong connectivity” further cements his academic influence as a core researcher in the NIBS-AD field.

### 3.4 Analysis of document co-citation

**Figure 10:**
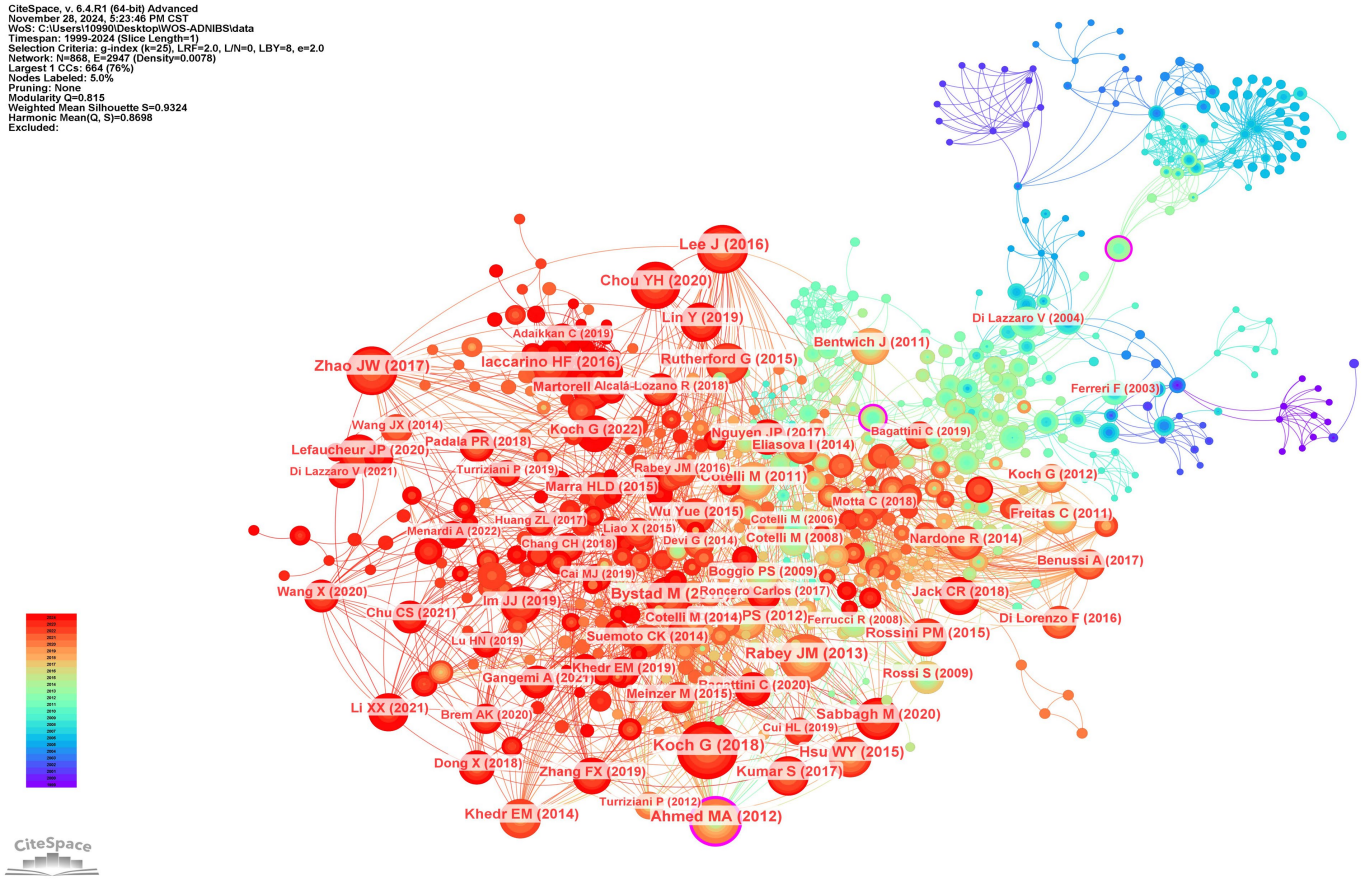
Documents co-citation network map.

**Figure 11:**
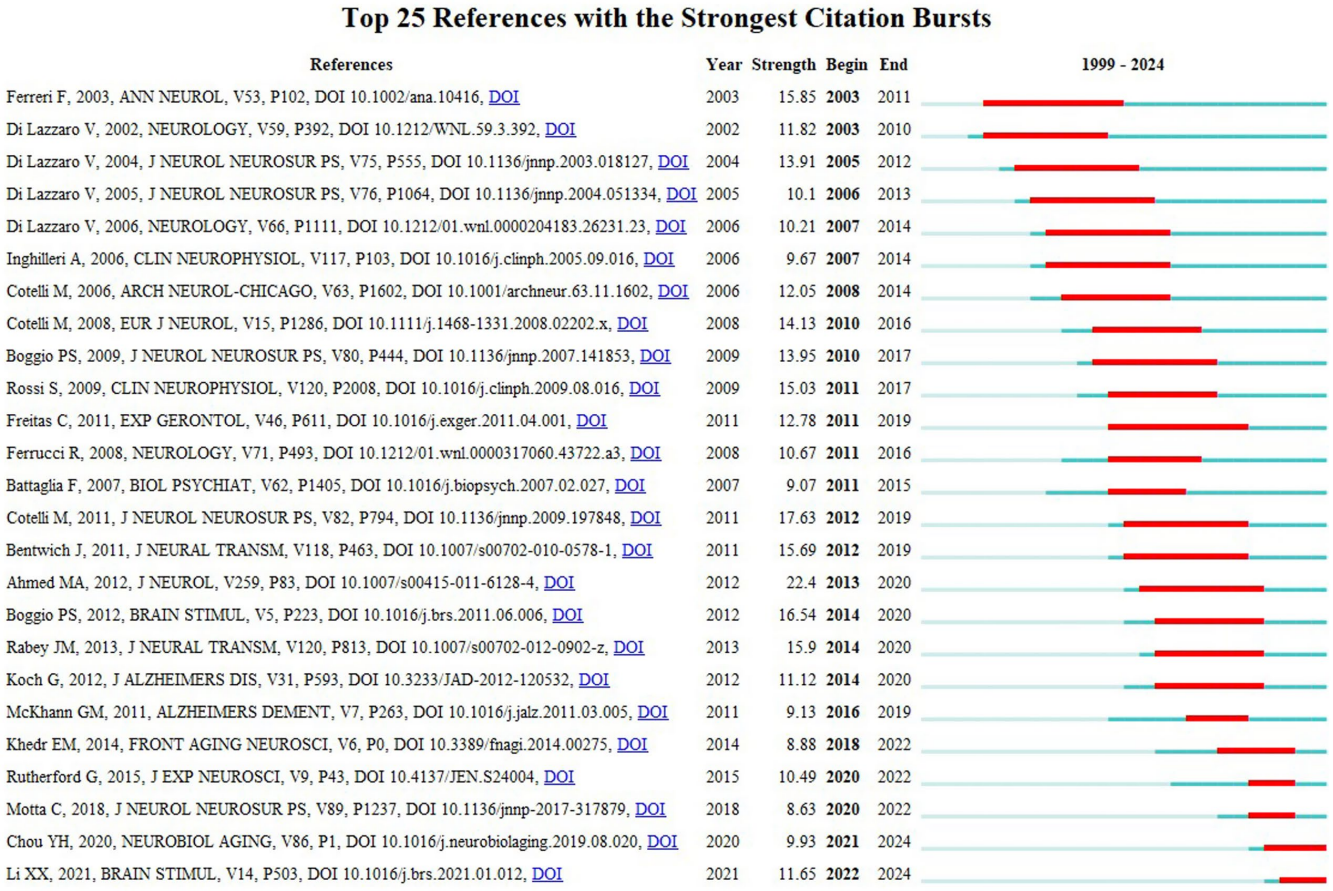
Citation burst detection diagram of the top 25 cited documents.

Analysis of cited references (Figure 10) revealed that the article by Koch et al. (2018), titled “Transcranial magnetic stimulation of the precuneus enhances memory and neural activity in prodromal Alzheimer’s disease,” published in Neuroimage in 2018, was identified as the most cited (87 citations). This study demonstrates that high-frequency transcranial magnetic stimulation targeting the precuneus significantly enhances brain network connectivity and memory function in AD patients. Figure 11 presents the top 25 articles with the strongest citation bursts related to NIBS and AD, based on burst strength. The article by Cotelli et al. (2011), titled “Improved language performance in Alzheimer disease following brain stimulation,” published in Journal of Neurology Neurosurgery and Psychiatry, demonstrated the highest burst strength (17.63), with the burst period spanning from 2012 to 2019. This means that in the past eight years, this study has been the core literature in the field that has been discussed and cited. It has confirmed the improvement effect of brain stimulation technology on the key functions of AD patients such as language fluency and word retrieval, which provides support for the subsequent exploration of “cognitive function-specific intervention.” Two articles showed the longest burst duration of 8 years: “Motor Cortex Excitability in Alzheimer’s Disease: A Transcranial Magnetic Stimulation Study” by Ferreri et al. (2003), published in Annals of Neurology (burst strength = 15.85), through TMS revealed the abnormal characteristics of motor cortex excitability in AD patients, and provided a new perspective for understanding the neuroepiphysiological mechanism of the disease. And “Noninvasive brain stimulation in Alzheimer’s disease: systematic review and perspectives for the future” by Freitas et al. (2017), published in Experimental Gerontology (burst strength = 12.78), which clearly sorted out the potential and challenges of NIBS in AD intervention, and provided an important theoretical framework for the subsequent research direction in this field. Another study by Chou et al. (2020) published in Neurobiology of Aging titled “A Systematic Review and Meta-Analysis of rTMS Effects on Cognitive Enhancement in Mild Cognitive Impairment and Alzheimer’s Disease,” provided more compelling evidence-based support for TMS’s cognitive improvement effects by synthesizing multi-center clinical data. Additionally, Li et al. ‘s study (Li et al., 2021) in Brain Stimulation titled “Cortical plasticity is correlated with cognitive improvement in Alzheimer’s disease patients after rTMS treatment” delved into the core mechanism of NIBS: exploring the relationship between cortical plasticity and cognitive function enhancement, offering crucial theoretical foundations for optimizing treatment protocols. Both studies remain actively cited as of 2024. This analysis not only reveals the hot research topics and their temporal evolution trends, but also provides guidance for future research focusing on the precise stimulation program of core brain areas and in-depth exploration of long-term efficacy and mechanism.

### 3.5 Analysis of keywords

**Figure 12:**
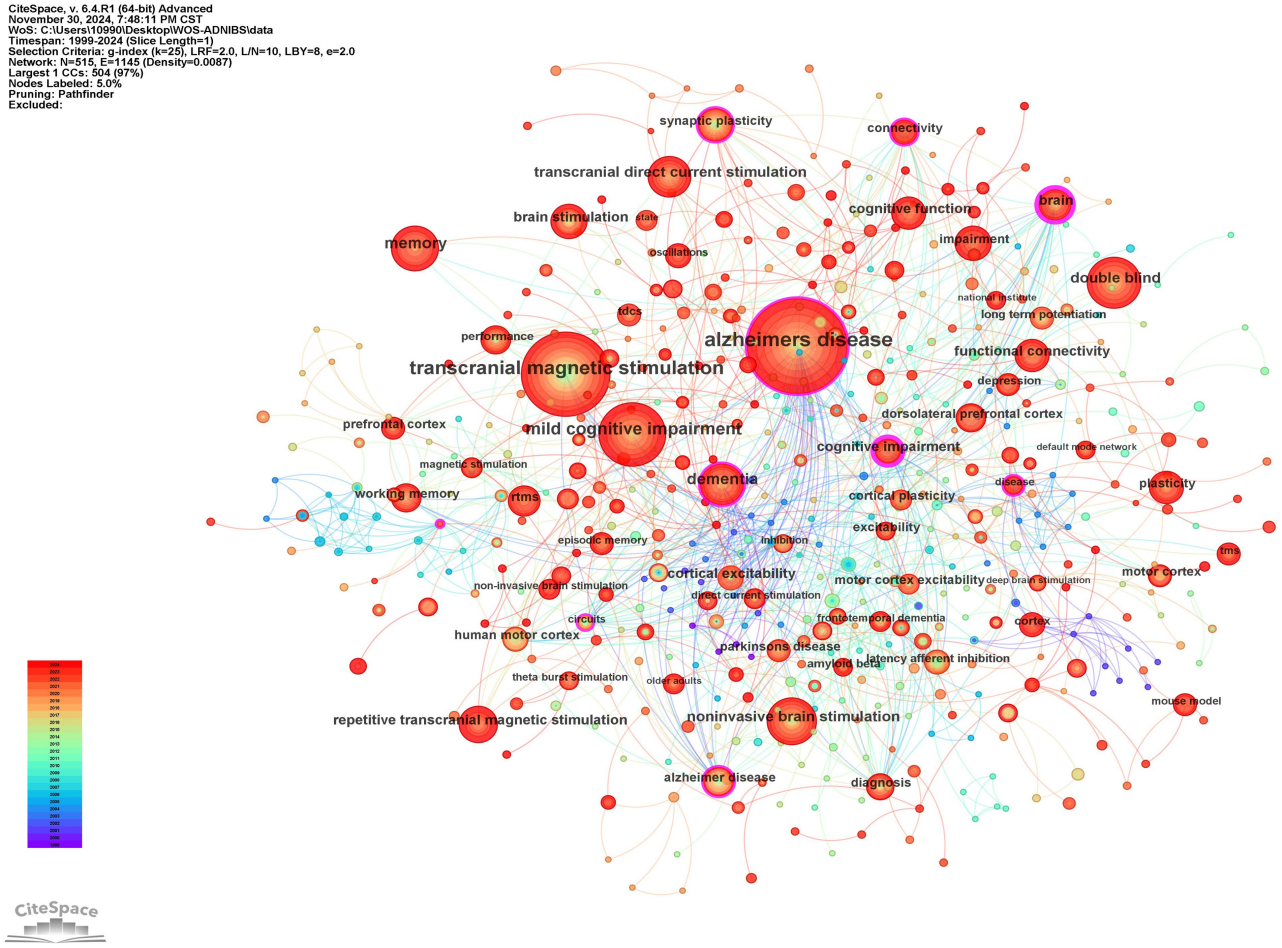
Keyword co-occurrence map.

Figure 12 illustrate the keyword co-occurrence network in this study. The most frequent keywords were “Alzheimer’s disease” (378 occurrences), “transcranial magnetic stimulation” (284 occurrences), “mild cognitive impairment” (161 occurrences), “memory” (92 occurrences), and “dementia” (92 occurrences). And the color change of the line between the keywords represents the time change of their respective occurrence, and the color change from bottom to top represents the distance of the research time. The red line indicates the recent trend. Over time, research trends have evolved significantly. Notably, the diversification of keywords related to NIBS has expanded the scope of research topics, with TMS, tDCS, tACS, and synaptic plasticity continuing to dominate the research landscape in this field.

**Figure 13:**
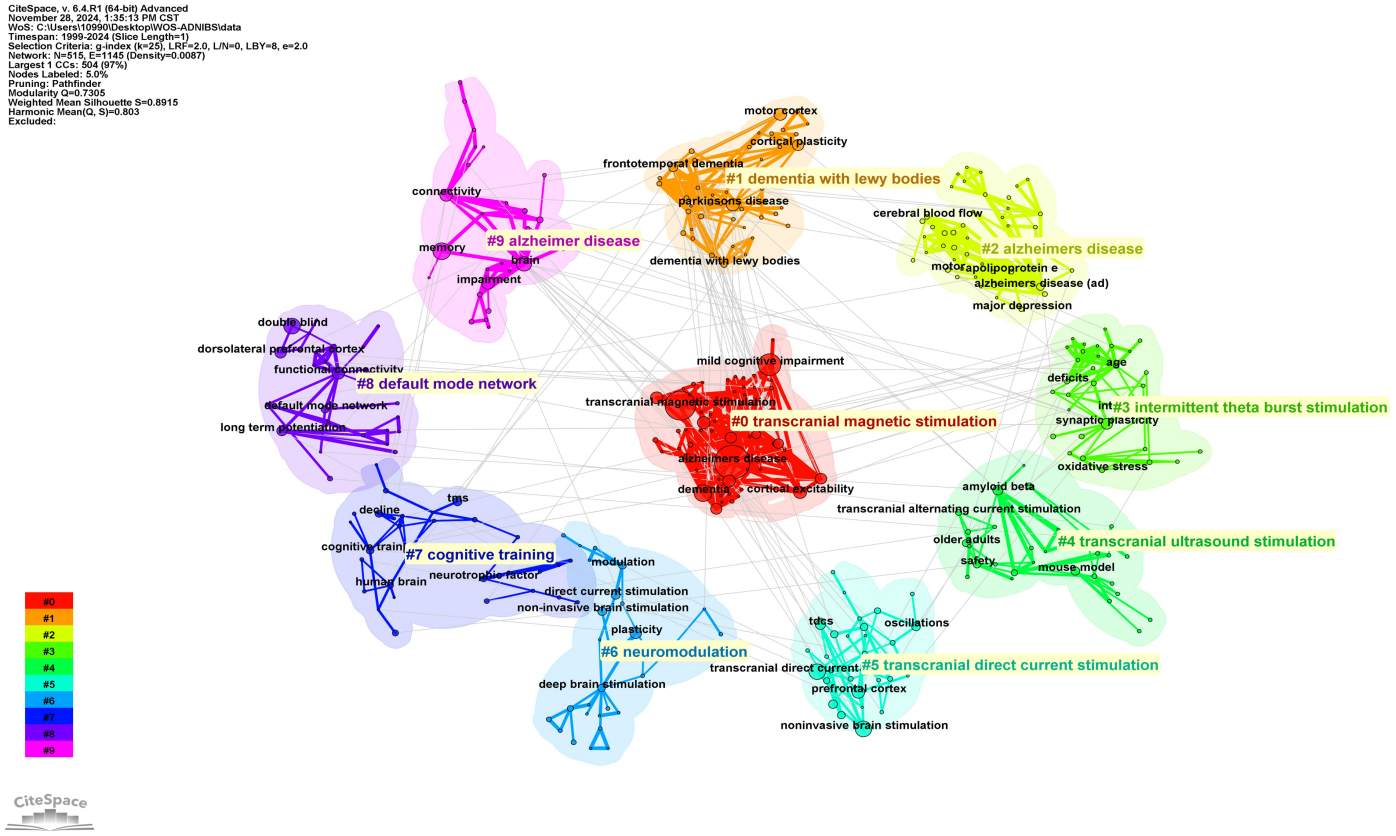
Keyword co-occurrence cluster diagram.

Figure 13 illustrates the thematic clusters formed by keywords. The red cluster centered on “transcranial magnetic stimulation” represents the largest research sector within this network. Significantly, the top three keywords in this cluster perfectly align with those most frequently identified in comprehensive analyses, highlighting their central role in the research landscape. Moreover, the 10 clusters are roughly divided into three directions: neurodegenerative diseases, theory and application of NIBS technology, intervention methods and mechanisms, reflecting the multi-level research pattern of “technology development—disease application—mechanism exploration” in the field.

**Figure 14:**
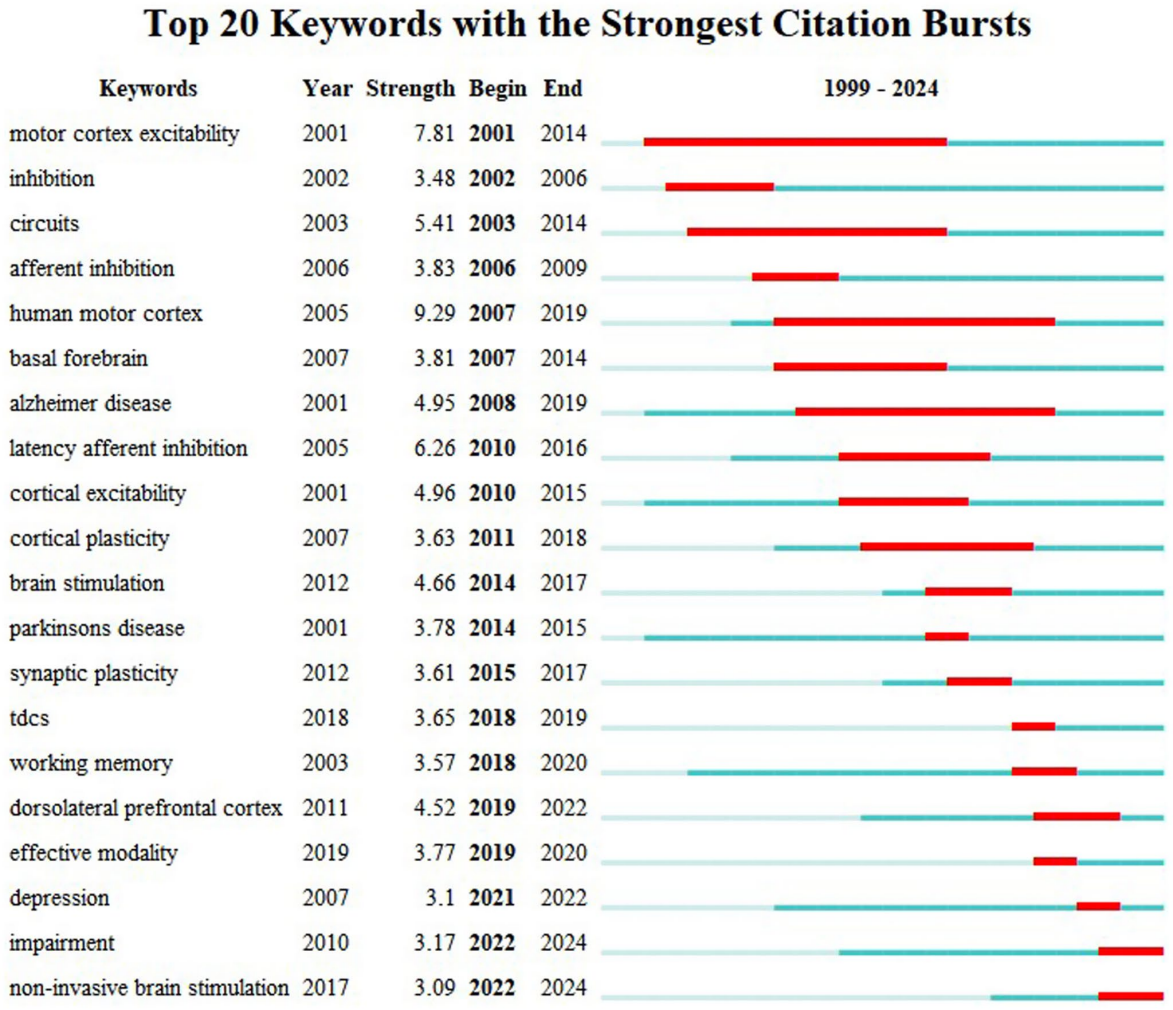
Burst detection diagram of the top 20 keywords.

Figure 14 presents the top 20 burst keywords based on burst strength. The keyword “human motor cortex,” which emerged in 2005, demonstrated the highest burst strength (9.29), with its burst activity spanning from 2007 to 2019. This was followed by “motor cortex excitability,” which appeared in 2001 and demonstrated the longest burst duration (13 years) with a burst strength of 7.81. Notably, “impairment” and “non-invasive brain stimulation” emerged as burst keywords in 2022 and continue to exhibit burst activity to the present. This analysis provides a deeper understanding of the evolution of research hotspots and facilitates the identification of future research frontiers.

## 4 Discussion

This study analyzed research articles related to the specific applications of NIBS in AD from the establishment of the WOSCC database up to November 27, 2024. Data analysis and visualization were conducted using Microsoft Excel, CiteSpace, GraphPad Prism, VOSviewer, and NoteExpress software to facilitate a comprehensive and visually accessible understanding of the current research landscape, hotspots, and emerging trends in the application of NIBS for AD. By contrasting previous analyses of single NIBS technologies, the results of this study fill the comprehensive application research gap in comprehensive NIBS application research for AD, and provide ideas for future mechanism research and clinical treatment in this field.

### 4.1 Research trends

Literature analysis shows that research related to NIBS-AD presents a generally upward trend, with the most significant growth rate during 2020–2021. Based on the analysis results of annual publication rankings, countries, institutions, and authors, the top three journals publishing NIBS-AD studies were Journal of Alzheimer’s Disease, Frontiers in Aging Neuroscience, and Clinical Neurophysiology. Multiple countries have contributed to this field, with Italy, China, and the United States leading in publication output. Among them, Italy and the United States rank first in the world in terms of the number of papers published and have high citation centrality, indicating that they occupy a leading position in promoting NIBS-AD research. Italy’s leading position is closely related to its long-term research accumulation, continuous investment from key institutions (such as Sapienza University of Rome, Univ Roma Tor Vergata), and academic cooperation networks of professionals (such as Giacomo Koch, Martorana, A, Di Lorenzo, F). China started relevant research in 2010, and the number of papers has increased sharply. However, the citation frequency of these achievements is relatively low, which may be related to the high proportion of self-citation patterns and language differences. The co-occurrence maps of countries, institutions, and authors show that the global cooperation network has been increasingly strengthened in recent years. To further promote in-depth research on NIBS-AD, interdisciplinary collaboration between leading institutions in various countries is crucial. At the same time, China should strengthen exchanges and cooperation with top international institutions to improve its academic influence in this field.

Combined with high-frequency keywords, keyword clustering, and keyword burst detection, within the analyzed time range, “motor cortex excitability” maintained the longest duration of research interest (13 years, 2001–2014), while “human motor cortex” demonstrated the highest burst strength (9.29). Over time, the research focus has shifted from early exploration of basic physiological functions such as “human motor cortex” and “motor cortex excitability” to discussions on the expanded application of non-invasive technologies, as well as disease-related intervention measures and mechanisms. For example, the targeted intervention of AD using technologies like TMS and tDCS, and their improving effects on neuroplasticity, cerebral blood flow and metabolism, neuroinflammation, and brain networks. Current bibliometric analysis indicates that while numerous NIBS-AD clinical trials prioritize immediate cognitive metrics, critical evidence gaps remain regarding long-term treatment sustainability. Cluster analysis reveals fundamental fragmentation in mechanistic research. As shown in Figure 13, clusters labeled “neuromodulation” (Cluster #6), “cognitive training” (#7), and “default mode network” (#8) demonstrate lower connectivity compared to the overall dataset, reflecting persistent gaps between preclinical and clinical evidence validation in neuroplasticity research. The emergent keywords “impairment” and “non-invasive brain stimulation” in 2022 mark the official shift of research focus to “pathological injury repair” and “multimodal technology integration,” providing new targets for Phase III clinical trials, and ultimately forming a research and treatment model of “basic mechanism—technology research and development—disease application—mechanism re-exploration.”

### 4.2 Stimulation modalities

NIBS exhibits therapeutic potential for AD through mechanisms that enhance neuroplasticity, modulate neurotransmitter levels, and regulate cerebral blood flow dynamics. Keyword co-occurrence and burst detection analyses reveal that TMS and tDCS constitute the predominant NIBS modalities for AD, with transcranial alternating current stimulation (tACS) emerging as an additional clinically validated approach. TMS generates a magnetic field that directly influences neurons, inducing action potentials and thereby adjusting neural circuit functionality. This process promotes neuroplasticity, as well as synaptic plasticity. On the other hand, tDCS modulates the resting membrane potential, thereby altering neuronal excitability and fostering neural regeneration. Furthermore, research indicates that NIBS can enhance the production of neurotransmitters such as Brain-Derived Neurotrophic Factor (BDNF), increase local glucose metabolism, and regulate neuronal excitability, collectively contributing to the amelioration of cognitive functions and behavioral symptoms in AD patients (Chen et al., 2019; Pang and Shi, 2021; McNerney et al., 2022; Cocco et al., 2018).

By acting on the cerebral cortex, rTMS can not only inhibit compensatory hyperactivation, but also maintain cortical plasticity. Evidence indicates superior efficacy of HF-rTMS over low-frequency rTMS (LF-rTMS) under equivalent stimulation parameters (Kim S. K. et al., 2024; Kim T. et al., 2024; Lanni et al., 2024). In addition, single-pulse TMS testing targeting the primary motor cortex can generate evoked potentials (MEPs) for the assessment of cortical excitability and neuroplasticity (Ferreri et al., 2003; Wang et al., 2016; Di Lazzaro et al., 2021). A meta-analysis has revealed that in AD patients, reduced cognitive performance is significantly associated with increased cortical excitability, diminished cortical inhibition, and more severe impairments in cortical plasticity (Chou et al., 2022). Early detection of MEP-related indicators may therefore contribute to early diagnosis. However, it should be noted that TMS can detect changes in cortical excitability earlier than the emergence of typical pathophysiological features, though further pathological or blood tests are required for confirmation.

tDCS also demonstrates significant therapeutic efficacy in Alzheimer’s disease (AD) management (Hansen, 2012), Clinical evidence indicates that anodal tDCS (A-tDCS) selectively enhances recognition memory in AD patients, with single-session intervention improving Word Recognition Task (WRT) accuracy by 17% (Ferrucci et al., 2008). Boggio et al. also corroborated these findings, revealing that left dorsolateral prefrontal cortex (DLPFC) A-tDCS significantly boosts visual recognition memory and partially mitigates executive function decline (Boggio et al., 2008). Another study by the team found that after five consecutive sessions of A-tDCS on the temporal lobe cortex of AD patients, their performance in the visual recognition memory test significantly improved. This once again demonstrated the main effect of A-tDCS on improving memory performance (Boggio et al., 2012). Furthermore, functional MRI (fMRI) analyses revealed enhanced connectivity between the default mode network (DMN) and frontoparietal network (FPN) post-intervention, suggesting A-tDCS-induced reorganization of large-scale brain networks critical for cognitive processing (Boudewyn et al., 2019; Choe et al., 2016; Nissim et al., 2019). On the basis of the effectiveness of A-tDCS, clinical studies should be carried out to explore the best treatment mode of A-tDCS by investigating different stimulation frequencies and different intervention timing after onset.

Transcranial alternating current stimulation (tACS) interventions revealed compensatory adjustments in the default mode network (DMN) and frontoparietal network (FPN)—core networks exhibiting pathological hyperactivity and hypoconnectivity in AD patients. Neural oscillations, defined as rhythmic electrical fluctuations generated by synchronized neuronal populations, are categorized by frequency bands including *α* (8–12 Hz), *β* (15–30 Hz), and *γ* (30–100 Hz) oscillations. Preclinical evidence from AD mouse models indicates that chronic tACS administration significantly improves Y-maze performance through γ-oscillation entrainment, concomitant with reduced amyloid-β plaque burden (Wu et al., 2022). Notably, 40 Hz frequency-specific tACS achieves optimal γ-band entrainment and memory enhancement, as demonstrated across multiple experimental paradigms (Adaikkan et al., 2019; Martorell et al., 2019; Chan et al., 2022; Cimenser et al., 2021). The corpus callosum, serving as the principal commissural pathway for interhemispheric integration, shows accelerated atrophy in AD that disrupts neural connectivity (Hofer and Frahm, 2006). γ-Oscillation induction via tACS exhibits neuroprotective effects by mitigating synaptic loss progression and decelerating corpus callosum degeneration rates (Traikapi and Konstantinou, 2021; Kasten and Herrmann, 2017). These findings collectively position γ-focused tACS protocols as promising disease-modifying strategies for preserving structural and functional connectivity in prodromal AD.

Taken together, these results establish HF-rTMS and A-tDCS as effective treatment modalities for AD to a certain extent, while other stimulation approaches should be supplemented by more clinical trials to prove them.

### 4.3 Therapeutic targets

Bibliometric analysis revealed Giacomo Koch’s 2018 article “Transcranial magnetic stimulation of the precuneus enhances memory and neural activity in prodromal Alzheimer’s disease” as the most-cited publication (Koch et al., 2018). The precuneus (PC), located in the parietal lobe, plays a crucial role in memory retrieval and serves as a key node in the default mode network. Research findings indicate that HF-rTMS targeting the PC offers a promising non-invasive solution for managing memory dysfunction in early-stage Alzheimer’s disease patients. This groundbreaking study identifies the PC as a vulnerable transitional zone during dementia progression and proposes it as an ideal target for personalized interventions to address AD-related memory decline. At the same time, PC-targeted rTMS reduces compensatory hyperactivation while preserving cortical plasticity, with HF-rTMS specifically enhancing episodic memory performance (Ge et al., 2023; Koch et al., 2022). A randomized controlled trial also confirmed that PC-directed HF-rTMS strengthens PC-hippocampal connectivity, yielding broad and sustained cognitive improvements, particularly in episodic memory domains (Traikapi et al., 2023).

It has been found that AD rTMS treatment usually targets the dorsolateral prefrontal cortex (DLPFC) and the precuneus (PC). The DLPFC, the most frequently targeted region in AD-related rTMS research, while DLPFC plays pivotal roles in higher cognitive functions including cognitive control, attention, working memory, and episodic memory (Hall et al., 2024; Xie et al., 2021). Ahmed et al. demonstrated that AD patients receiving DLPFC-targeted HF-rTMS exhibited enhanced Mini-Mental State Examination (MMSE) scores, improved linguistic performance, and superior task accuracy (Aloizou et al., 2021). While bilateral rTMS stimulation enhances functional connectivity within brain networks, left-sided DLPFC HF-rTMS demonstrates particular efficacy in global memory enhancement (both immediate and long-term) through neurotransmitter level modulation (Sharbafshaaer et al., 2023; Bouaziz et al., 2021; Xiu et al., 2024; Baeken et al., 2011). tDCS targeting DLPFC also showed excellent therapeutic effects. Multiple studies have demonstrated that anodal transcranial direct current stimulation (A-tDCS) targeting the left DLPFC ameliorates cognitive impairments in AD patients, including visual and verbal recognition memory deficits, and exerts significant neuroprotective effects (Boggio et al., 2012; Ferrucci et al., 2008; Šimko et al., 2022). A study by Li XX et al. demonstrated that twice-daily A-tDCS targeting the left DLPFC combined with cathodal tDCS (C-tDCS) over the right DLPFC modulated cortical excitability, potentially reversing age-related neuroplasticity impairments and enhancing cognitive performance in elderly patients (Li et al., 2023).

Clinical evidence has demonstrated that targeting the DLPFC and PC through stimulation holds particular promise. However, given significant individual variations among patients, future research should focus on optimizing stimulation targets and protocols while exploring multi-target synergistic therapies to maximize treatment efficacy. It is necessary to further integrate multimodal diagnostic techniques such as functional magnetic resonance imaging (fMRI), functional near-infrared spectroscopy (fNIRS), and electroencephalography (EEG) to identify optimal therapeutic targets for AD.

### 4.4 Limitations

This study has several limitations. Firstly, the literature search was restricted to the WOSCC database and included only English-language studies, potentially leading to the omission of relevant studies and the absence of a systematic quality assessment of the included literature. Secondly, we observed that the problem of proper noun standardization (e.g., “Alzheimer’s disease” and “Alzheimer disease”) was not resolved during the analysis due to methodological limitations inherent in the bibliometric workflow. The direct importation of raw data from WOSCC without pre-processing normalization, coupled with the inability of analytical tools (CiteSpace and VOSviewer) to perform automated synonym resolution, may have compromised analytical accuracy through inconsistent term aggregation. Additionally, no detailed statistical analysis was conducted to compare specific stimulation techniques, and there is limited exploration of the clinical applicability and adoption rates of each method. Future research should prioritize large-scale, multi-center clinical trials, incorporate a broader range of literature. Moreover, the key words for proper nouns should be selected according to the international unified MeSH terms, and perform comprehensive analyses of their outcomes.

## 5 Conclusion

This study provides a comprehensive overview of the research overview, hotspots, and development trends in the application of NIBS for AD. We found that the current research hotspots predominantly focus on the effects of various NIBS techniques on cognitive function in AD, the selection of optimal treatment targets, and the underlying mechanisms. Based on the findings, this field is experiencing significant attention and rapid growth. To further advance the field, enhanced global collaboration among countries, institutions, and researchers is essential to drive deeper scientific and clinical progress.

## Data Availability

The original contributions presented in the study are included in the article/Supplementary material, further inquiries can be directed to the corresponding authors.
