# Correction: Current application status of non-invasive brain stimulation techniques in Alzheimer's disease: a bibliometric analysis

**DOI:** 10.3389/fnagi.2025.1730649

**Published:** 2025-11-17

**Authors:** Shan Cong, Meng Wang, Long Yan, Li Sun, Bowen Zheng, Jinying Xie, Tao Yu, Yulin Qian

**Affiliations:** 1 The First Teaching Hospital of Tianjin University of Traditional Chinese Medicine, Tianjin, China; 2 National Clinical Research Center for Acupuncture and Moxibustion, Tianjin, China; 3 Graduate Department, Tianjin University of Traditional Chinese Medicine, Tianjin, China; 4 Tianjin Xiqing District Traditional Chinese Medicine Hospital, Tianjin, China

**Keywords:** Alzheimer's disease, non-invasive brain stimulation, transcranial magnetic stimulation, bibliometric analysis, CiteSpace

In the published article, there was an error in the **Funding** statement. An incorrect **Funding** source was provided: [the Tianjin Municipal Education Commission Research Plan Project (Natural Science) (no. 2024KJ024)].

The corrected **Funding** statement appears below.

**Funding**

The author(s) declare that financial support was received for the research and/or publication of this article. This study was supported by the Science & Technology Development Fund of Tianjin Education Commission for Higher Education (No. 2024KJ024).

The original version of this article has been updated.

